# Risk perception in fire evacuation behavior revisited: definitions, related concepts, and empirical evidence

**DOI:** 10.1186/s40038-014-0005-z

**Published:** 2015-01-08

**Authors:** Max T Kinateder, Erica D Kuligowski, Paul A Reneke, Richard D Peacock

**Affiliations:** 1 Department of Cognitive, Linguistic, and Psychological Sciences, Brown Unversity, Providence, RI USA; 2 National Institute of Standards and Technology, Gaithersburg, MD USA

**Keywords:** Risk perception, Egress, Evacuation, Evacuation modeling, Fire safety, Human factors

## Abstract

Risk perception (RP) is studied in many research disciplines (e.g., safety engineering, psychology, and sociology). Definitions of RP can be broadly divided into expectancy-value and risk-as-feeling approaches. In the present review, RP is seen as the personalization of the risk related to a current event, such as an ongoing fire emergency; it is influenced by emotions and prone to cognitive biases. We differentiate RP from other related concepts (e.g., situation awareness) and introduce theoretical frameworks relevant to RP in fire evacuation (e.g., Protective Action Decision Model and Heuristic-Systematic approaches). Furthermore, we review studies on RP during evacuation with a focus on the World Trade Center evacuation on September 11, 2001 and present factors modulating RP as well as the relation between perceived risk and protective actions. We summarize the factors that influence perception risk and discuss the direction of these relationships (i.e., positive or negative influence, or inconsequential) and conclude with presenting limitations of this review and an outlook on future research.

## Introduction

**Figure 1:**
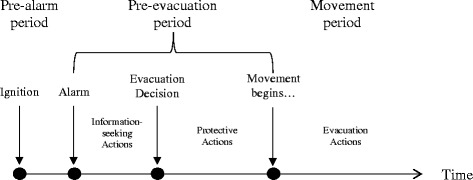
**Timeline of building fire evacuation.**

Occupants need to reach a place of safety during building fire emergencies. Evacuation behavior enables building occupants to do so (ISO/IEC 2008). Figure 1 gives an overview of the evacuation process and it illustrates that occupant evacuation from buildings comprises two distinct periods: *pre-evacuation* and *evacuation period* (Kuligowski et al. 2010). The pre-evacuation period can be further split into a pre-alarm phase, an information seeking phase, and a response phase (in which initial protective actions are taken); it ends when an evacuation decision is made (Purser and Bensilum 2001). The crucial point in the pre-evacuation period is the decision of occupants to evacuate after they have received initial fire cues^a^, which marks the transition from pre-evacuation to evacuation behavior. This decision is potentially dependent on occupants’ risk perception (RP) and other human factors. For a recent review of human factors in building evacuation, see Ronchi and Nilsson (2013).

Engineering tools such as evacuation computer models, aim to establish the Available/Required Safe Egress Time (ASET/RSET) of a building. RSET is defined as the time occupants need from the fire onset until they reach a place of safety. ASET refers to the time which is actually available for evacuation, specifically the time between the onset of a fire and the point at which conditions become fatal (Kobes et al. 2010). Most evacuation models implement oversimplified assumptions about the pre-evacuation period. For example, psychological processes and social interactions are often not considered. For an overview, see Kuligowski et al. (2010) or Proulx (2008). This increases the uncertainty in evacuation models, as studies have shown that the pre-evacuation period can be as long as or longer than the actual evacuation time period (Proulx 1995; Fahy and Proulx 2001; Kobes et al. 2010).

It is important to understand RP during building fire evacuations for many reasons. First, since RP is hypothesized to influence the point of transition from pre-evacuation to evacuation (or other protective) behavior, it is questionable whether an accurate description of the evacuation process is possible in the absence of an accurate description of the RP. In the worst case, faulty assumptions about RP may find their way into evacuation models or affect building design. Second, understanding RP and its relevance for evacuation decision-making may in turn contribute to the development of more accurate evacuation models, via more precise predictions of ASET/RSET, and ultimately improve building safety. A significant part in this endeavor is the eventual development of a comprehensive behavioral theory on human behavior in fire (Kuligowski and Gwynne 2010). A comprehensive theory of human behavior in fire would describe and explain aspects of evacuation behavior in logical terms that are consistent with systematic observations of the real world.

A review of the literature on the topic of RP has highlighted a variety of ways in which RP has been discussed. First, research studies on the topic often attempt to identify the factors that influence perceived risk. These factors can be individual-based (i.e., psychophysiological states or traits of an individual), physical (i.e., in the environment) or social (i.e., the behavior of others) in nature. Second, research studies have questioned whether RP influences aspects of the evacuation process such as the evacuation decision or evacuation delay time (i.e., the time occupants wait before they start evacuating). In either case, literature on RP and evacuation often does not propose a definition of RP or the way in which the research has defined the term (See Table 1 for an overview of different ways of operationalization of perceived risk in research studies). Therefore, the first goal of this literature review is to clarify the concept of RP in the context of building fire evacuation, and to provide a definition of RP specifically for the field of fire protection engineering. This includes the distinction from similar relevant concepts (e.g., situation awareness) and a discussion of the scope (e.g., the spatial and temporal proximity of a threat) of RP is presented.

**Table 1:** **Overview of studies on RP and evacuation**

**Ref.**	**Rel.** ^**3**^	**Scenario**	**N**	**Study population**	**Transfer to building fires possible?** ^**2**^	**Qual./Quan.**	**Control group**	**Data**	**Method**	**Measure of RP**	**Theory**	**Factors affecting RP**	**RP related to evacuation** ^**3**^
Kuligowski and Mileti (2009)	3	Building evacuation under a terrorist attack	803	WTC occupants^**1**^	yes	Quan.	no	Retro-spective		1 item (yes/no): “During the time when you first became aware that something had happened and when you first entered the stairwell or elevator to leave did you believe that other people were in danger of being killed?”	PADM	Environmental cues, floor level, obtained information,	No direct effect on evacuation delay (beta ≈ 0 for both towers); weak effect on information seeking behavior ( beta ≈ 0.15) in one tower, pre-evacuation actions were associated with higher perceived risk (beta ≈ 0.23 vs. beta ≈ 0.08).
Day et al. (2013)	3	Building evacuation under a terrorist attack	240	WTC occupants^**5**^	yes	Quan.	no	Retro-spective	Interview	7 point Likert scale (“How much at risk did you feel”)	-	Number of cues, quality of cues, distance to impact	High perceived risk predicted early responders (Beta = .36; OR = 1.44) Low perceived risk was not a predictor of delayed evacuation
Kuligowski (2011)	3	Building evacuation under a terrorist attack	252	WTC occupants^**5**^	yes	Qual.	no	Retro-spective	Interview	7 point Likert scale	PADM	Previous experience, hyper vigilance, cue intensity, cue identification	Perceived risk predicted evacuation decision
Sherman et al. (2011)	3	Building evacuation under a terrorist attack	1139	WTC occupants	yes	Quan.	no	Retro-spective	Question-naire	1 item asking “How serious did you think the situation was at first?” on a 4 point Likert scale	-	female, member of port authority NY/NJ, personal background variables; evacu-atingfromWTC1 (vs WTC2), more Environmental Cues, more unusual Events (context variables), lower education, longer tenure in the towers, more knowledge, more emergency preparedness	lower perceived risk:
													- less information seeking
													- more pre-evacuation actions
													- longer pre-evacuation delays (beta = −.25)
Gershon et al. (2007)	3	Building evacuation under a terrorist attack	50	WTC occupants	yes	Qual.	no	Retro-spective	In-depth Interviews (n = 30) or focus groups (n = 20)	Coding of qualitative interviews	-	-	Yes, emergent perception of risk formed by sensory cues facilitated evacuation decisions (but not the process of evacuation)
Gershon et al. (2012)	3	Building evacuation under a terrorist attack	1444	WTC occupants	yes	Qual.	Comparison to WTC occupants who were not in the building at the incident	Retro-spective	Questionnaire	Several items (number not specified), including seriousness of the situation, and concerns that the building would collapse	Behavioral Diagnostic Model	-	Yes, 70% stated that they evacuated because they appraised the situation as dangerous. Occupants who thought the situation was serious evacuated with less delay (OR = 3.78) and faster (OR = 1.80).
Caroly et al. (2013)	3	Tunnel accident and fire	11 tunnel fires	Tunnel users	With limitations	Qual.	no	Retro-spective	Review of reports, video footage, media reports	Not reported	Danger control model	Visibility of cues	Yes
Averill et al. (2012); Averill et al. (2007)	2	Building evacuation under a terrorist attack	400	WTC occupants^**1**^	Yes		no	Retro-spective	Interviews			Seek info, environmental cues	
McConnell et al. (2010)	2	Building evacuation under a terrorist attack	126	WTC occupants^5^	yes	Quan.	no	Retro-spective	Questionnaire	7 point Likert scale	-	Floor level in tower, WTC1, time (before or during evacuation)	-
Jönsson et al. (2012)	2	Elevator evacuation during an unspecified emergency	573	High-rise building occupants	Yes, with limitations	Quan.	no	Cross-sectional	Hypothetical scenario questionnaire	Rating of perceived safety of evacuation routes (two 7 point Likert scale items)	-	Building floor, evacuation method (elevator vs. staircase)	yes
Mbaye and Kouabenan (2013)	1	Accident in chemical/nuclear facility	302	Employees in chemical & nuclear facility	With limitations	Quan.	no	Cross-sectional	Questionnaire		-	locus of control, positivity bias, availability heuristic	-
Riad et al. (1999)	1	Hurricane evacuation	777	Residents in hurricane risk regions	With limitations	Quan.	no	Retro-spective	Interview		-	-	yes
Brenkert-Smith et al. (2013)	1	Wildfire evacuation	747	Wildland-urban interface (WUI) homeowners in Boulder and Larimer Counties in Colorado, USA	Yes, with limitations	Quan.	No	Prospective	Questionnaire	2 questions on perceived probability scaled to range from 0 to 100 and Likert scale for 4 variables on perceived consequences	Social amplification of risk framework	Lot size, Previous experience, social context	-
Lindell et al. (2005)	1	Hurricane evacuation	206-407	General population in hurricane area	With limitations	Quan.	no	Retro-spective	Questionnaire	-	-	-	-
Matyas et al. (2011)	1	Hurricane evacuation	448	Tourists	With limitations	Quan.	no	Cross-sectional	Questionnaire	5 point Likert scale	-	-	Yes (correlated with stated preference)
Horney et al. (2010)	1	Hurricane evacuation	570	General public	With limitations	Quan.	no	Retro-spective	Interview	3 point scale (low-middle-high)	PADM	Actual risk, homeownership,	no
Martin et al. (2009)	1	Wildfire evacuation	251	Fulltime & seasonal residents	With limitations	Quan.	no	Retro-spective	Questionnaire	5 point Likert scale	PADM	Fire experience, subjective knowledge, perceived responsibility	Yes, mediated; 38% of variance in perceived risk explained
Siebeneck and Cova (2012)	1	Flood evacuation	196	General population in flood area	With limitations	Quan.	no	Retro-spective	Questionnaire	5 point Likert scale	Threshold model of RP	Distance to threat, Time course of events, amount of property damage	Not reported
Drabek (2001)	1	Natural disaster	406	Business employees	With limitations	Qual.	no	Retro-spective	Questionnaire	4 items measuring risk-related behavior and perceived safety	Stress–strain perspective	Higher perceived risk was associated with lower amount of community disaster planning, warning messages implying that evacuation was mandatory, residing in a mobile home or apartment, working in a more formalized company, working in a younger company, and long-term event or consequences	Perceived risk predicted evacuation delay (beta = .145) multiple evacuation (beta = .158)

*Note:* The content of this table is solely based on the information available in the individual studies and the amount and accuracy of the reported information varies. Ref. = Reference; Rel. = Relevance; N = sample size; Quan. = Quantitative study; Qual. = Qualitative study; WTC = World Trade Center; ^**1**^NIST WTC evacuation data base; ^**2**^yes, with limitations, no, unclear; ^3^If yes, describe the relation (e.g. mediated, correlated); ^3^ 1 = planned evacuation from a latent threat, 2 = acute evacuation from an acute threat than building fire, 3 = Fire evacuation from buildings; ^4^labeled as milling in this study; ^**5**^HEED data base; ^6^ no specification of actual number of participants was given in this paper.

The studies are sorted according to their relevance for RP and evacuation.

When studies on RP are presented, researchers have often identified some theoretical underpinning of risk perception that provides the foundation for the methods in the study. Thus, the second goal of this review is to identify and describe relevant theoretical frameworks of RP from evacuation research and other disciplines.

The third goal is to present a systematic overview, summary and discussion of the factors affecting RP during fire evacuation. Specifically, the current knowledge on the role of RP in the pre-evacuation and evacuation period in the fire evacuation process as well as factors modulating the relation between RP and protective actions are discussed. This way, the present overview may contribute to theory development in fire evacuation research.

## Methods

For the purpose of the present literature review, we followed the steps for a systematic literature review suggested by Khan et al. (2003):

Step 1 Framing questions for a review: The following main research questions were formulated: What is RP? And what role does RP play during building fire evacuation? These questions comprise the headings for the main chapters of this review. Each of these two very broad questions was subdivided into several steps which represent the sub headings in the each chapter.
Step 2 Identifying relevant work: Relevant literature on RP was primarily identified by searching literature data-bases (Web of Science, Google Scholar, Science Direct, Social Science Research Network, EvacMod.net). The keywords used to identify relevant literature included the following terms: *risk perception, evacuation, fire emergencies, human factors, human behavior in fire, hazard perception, egress, disaster, situation awareness, threat awareness, risk assessment, perceived vulnerability, arousal, risk communication, safety climate, safety culture, hurricane evacuation, heuristics, systematic information processing,* and *decision-making.* The sources were accessed through the libraries of the National Institute of Standards and Technology and the University of Würzburg, Germany. For literature without full text access from either of these two libraries or through interlibrary loan, abstracts were considered, or the source was ignored. The literature identified included but was not limited to reports and journal articles from fire research, psychology, sociology, and biology. The search results were integrated with relevant literature from colleagues and other publications.
Step 3 Assessing the quality of studies: Literature was included if it was relevant to the topic. Only publications in peer reviewed journal articles, conference proceedings, or books from established scientific publishers were considered. The literature research was not restricted to a certain time period, journal, field, or geographical location. An important criterion was the precision of the description of study protocol, sample, data collection, and analysis methods. Since studies from a variety of fields were included at this point, studies were ranked according to their relevance to RP and fire evacuation (Table 1).
Step 4 Summarizing the evidence: The main findings of the first question (What is RP?) are summarized in text in Section What is RP? Defining RP during fire evacuation. The results for the second question (What is the role of RP during fire evacuation?) are summarized in text and tables in Section What role does perceived risk play in building fire evacuation? and Section Overview of studies. The summaries address differences regarding the theoretical foundation, methods of data collection and analysis, as well as the interpretation of results of individual studies.
Step 5 Interpreting the findings: The methods, results, and their implications are discussed, followed by a discussion of the limitations of the present literature review (Section Limitations). Finally, future research questions for the topic of RP in the field of fire safety engineering are identified (Section Conclusions and outlook).

## What is RP? Defining RP during fire evacuation

### Definition of RP during fire evacuation

In the context of fire evacuation, RP refers to the perception of an imminent threat to one’s own life and health. Here, RP is defined as a psychological process that describes the subjective (conscious and unconscious) evaluation (as opposed to objective risk assessment) of the probability to be affected by an imminent undesirable event in a specific situation and an assessment of one’s own perceived vulnerability and coping resources. RP is seen as the process of personalizing the risk related to the current event, such as an ongoing fire emergency. It is influenced by emotions and prone to cognitive biases. Note that the term RP refers to a psychological process with *perceived risk* as its outcome. RP can be differentiated from several similar and overlapping concepts, such as situation awareness, perceived vulnerability, hazard perception, risk assessment and threat awareness (see Section Related concepts and expressions). Theoretical frameworks (Section Theoretical frameworks on RP and evacuation) provide an understanding on how risk relevant information processing and coping mechanisms translate into behavior in general.

Other scientific definitions of RP in a wider sense refer to the subjective assessment of the probability of an undesired event, the magnitude of its consequences, and one’s own coping capabilities (Michalsen 2003; Rayner and Cantor 1987; Wachinger et al. 2012). In this context, coping capabilities refer to general and situation specific competencies of an individual (e.g., the ability to stay calm in stressful situations or expertise in firefighting). There are two main approaches to RP. The first can be summarized as an *expectancy-value approach* (Patterson et al. 2010; Sjöberg et al. 2004) and the second can be referred to as the *risk-as-feelings* approach (Slovic 2010a).

According to the *expectancy-value approach*, RP consists of two components: an individual’s assessment of a natural hazard and his/her perceived vulnerability (Patterson et al. 2010). It comprises the beliefs (whether rational or irrational) held by an individual, group, or society about the likelihood, extent, magnitude, and timing of a threat; it refers to subjective assessments of probabilities of a specified type of accident happening, and how concerned one is with the consequences (Sjöberg et al. 2004). Here, RP is seen as a conscious cognitive process which is prone to biases. In the case of building fires, this would reflect an evaluation to the self-posed question “Am I at risk?” after having received fire cues (e.g., a fire alarm or smoke).

The *risk-as-feelings* hypothesis criticizes the assumption that RP is an (entirely) conscious cognitive process (Loewenstein et al. 2001; Slovic et al. 2005; Slovic 2010a). It stresses the role emotions play the moment decisions are made and it assumes that information needs to convey emotions in order to become meaningful for an individual. Here, RP refers to how much danger a person feels he/she is in as a result of the event (Sherman et al. 2011). For building fires, this would reflect an occupant’s “gut feeling” after perceiving fire cues.

Note that RP is seen as a subjective process of an individual. That is, RP is not necessarily related to objective risk (although potentially correlated) and is prone to various biases. One may hypothesize that both approaches are relevant and even connected for fire evacuation and simply refer to different aspects of how a building fire is experienced. Consequently, a holistic approach to RP in fire evacuation should include the expectancy-value as well as the risk-as-feelings approach.

The main difference between risk-as-feelings and the expectancy-value approach lies in the psychological processes, which may even be operating simultaneously (e.g., while walking through a dark empty street, one may feel at risk although one knows that one is in a safe area). Whereas the risk-as-feeling refers more to associative and emotional processes (“gut feeling”), the expectancy value process focuses on rational, cognitive processes. This differentiation is important for fire evacuation, since the results of the risk estimates of these processes can be different and consequently, behavior may vary depending on which approach is predominant.

### Characteristics of RP

As RP is studied in many research disciplines (e.g., fire protection engineering, psychology, and sociology) (Wachinger et al. 2012; Slovic 2000), the contexts to which concepts of RP are applied vary greatly. The following paragraphs elaborate on the characteristics of RP and links them to the specific case of fire evacuation.

As the term suggests, RP comprises a *risk* and a *perception* component. ‘Risk’ has various meanings in everyday usage, such as hazard (e.g., What are the most important risks for occupants during a building fire?), consequence (e.g., What is the risk of delayed evacuation during building fires?), probability (e.g., What is the risk of being in a building fire?), or potential adversity or threat (e.g., What is the risk of being exposed to a building fire?) (Slovic and Weber 2002). This highlights a critical aspect in many questionnaire studies on evacuation and RP in which participants were simply asked, ‘how much risk’ they felt (e.g., Day et al. 2013; Martin et al. 2009; Siebeneck and Cova 2012; Matyas et al. 2011; McConnell et al. 2010; Horney et al. 2010). It is possible that participants had different concepts about the term ‘risk’ when they rated their perceived risk. Note that these lay concepts of risk vary significantly from the scientific definition in fire safety, where risk is “the potential for realization of unwanted, adverse consequences to human life, health, property, or the environment. (Watts and Hall 2008, p. 3)”

‘Perception’ is defined as the organization, identification, and interpretation of sensory information in order to represent and understand the environment (Schacter et al. 2011). RP bridges all perceptive modalities and comprises various cognitive processes (e.g., sense-making, decision-making, or appraisal). In this context, perception can be understood as a signal-detection process, where occupants continuously scan their environment with their senses and have to filter threat-relevant fire cues from the noise of irrelevant input. This process results either in a hit (correct detection), miss (cue not detected/ incorrect rejection), false alarm, or ignore (correct rejection). The criterion as well as the signal-to-noise ratio affects the signal detection (See Green and Swets (1966) for an introduction to signal detection theory). Several factors, such as previous experience with fire, may lower the threshold criterion of detection or increase the sensitivity to fire cues (leading to more hits and false alarms). The more complex an environment becomes, the more the amount of sense-based “noise” increases, which makes it more difficult for an individual to differentiate fire cues from irrelevant stimuli (more misses). In other words, the fire cues become less salient for the occupants.

### Scope of RP research

The scope of RP research varies across disciplines and it is questionable if and how results can be transferred from one field to the other. In fact, RP studies on technological threats and natural hazards vary significantly in their outcome (Wachinger et al. 2012). Specifically, the scope of RP research can be described in a framework of threat certainty, the time frame of risks, and the target of RP.

*Threat certainty* refers to how likely an undesired event is and threats can be categorized into imminent or latent threats. Imminent threats are certain to occur, very near, or impending and require immediate responses. A latent threat refers to threats from potential disasters associated with certain risk factors, such as living in a high-risk hurricane or earthquake region. Here, the incidence of the actual event is not predictable (for an individual) in the foreseeable future. Most of the literature on RP during disasters covers latent threats in which consequences are uncertain, rare, and/or delayed. For emergency evacuation during fire, however, imminent threats are relevant.

The *time frame of risks* can be differentiated into short term and long term risks. Long term risks refer to risks that lie relatively far in the future (e.g., hurricanes that are near the coast for days in advance); for the present paper, short term risks lie in the immediate future (within hours, minutes or even less time). The long term perspective could be seen as the general tendency of a person to expect a threat. In the case of building fire evacuation, the time frame of risks is most likely short term.

The *target of RP* addresses the question of what is at risk for an individual. RP can be directed at various aspects of life; including one’s life and well-being, status, property, goals, and also others. For fire evacuation, the RP of one’s own life and health seems most relevant. This is also sometimes referred to as *personal risk* (Perry 1979). However, it is possible that other aspects of RP may compete with one’s own safety (Firing et al. 2009). For example, family members or significant others in a residential evacuation may act as a modulating factor for one’s own personal risk.

### Related concepts and expressions

The following section describes several concepts that either overlap with the present definition of RP or are sometimes used synonymously. Of these, *situation awareness* is the most relevant in the context of fire evacuation and will be discussed in more detail. Given the conceptual overlap, for example, in the importance of appraisal processes in RP and situation awareness, these related terms were also considered during literature research.

*Situation Awareness* (or sometimes known as situational awareness) is a key concept introduced by Endsley in the decision-making literature (Endsley and Jones 2013). Situation awareness is defined as “the perception of the elements in the environment within a volume of time and space, the comprehension of their meaning and the projection of their status in the near future” (Endsley 1988, p.97). It is conceptualized as an internalized temporal and spatial representation or mental model of a person operating in a complex environment (Endsley and Jones 2013; Endsley 1988; Sarter and Woods 1991). The quality and precision of such mental models affect decision-making and depend, among others, on the complexity of the environment. Poor situation awareness has been identified as a major cause in accidents related to human errors (Endsley 1995). Apart from the definition reported here, several other definitions can be found in the literature, of which a discussion is beyond the scope of this paper (See Sarter and Woods (1991) for a detailed summary of situation awareness concepts). Although closely related, situation awareness is a more holistic concept than RP as it applies to the environment as a whole and not only to hazards. Furthermore, situation awareness can be seen as a conscious process, whereas RP consists of conscious (expectancy-value assessments) and unconscious components (the feeling of risk). Some authors argue that RP can be understood as situation awareness for dangerous situations (Horswill and McKenna 2004). Although RP and situation awareness overlap, they are independent concepts. Individuals may feel at risk with both high and low situation awareness.

*Perceived vulnerability* is the subjective appraisal of one’s own capacity to anticipate, cope with, resist and recover from the impact of a natural hazard (Wisner 2004). Some authors use RP and perceived vulnerability synonymously (Riad et al. 1999).

*Hazard perception* is the skill to detect developing threats (McKenna and Crick 1991). This concept is mainly used in traffic research. Some authors argue that hazard perception reflects situation awareness for dangerous situations in the traffic environment and improves with training (Horswill and McKenna 2004).

*Threat awareness* (related *death awareness*) can be understood as the general mental model an individual has about life threatening events (Hirschberger et al. 2009). It is part of terror management theory, which addresses how humans cope with the idea of their own mortality (Greenberg et al. 1990).

*Risk assessment* is the “identification, evaluation, and estimation of the levels of risks involved in a situation, their comparison against benchmarks or standards, and determination of an acceptable level of risk” (Business Dictionary 2013). Most of the literature using this term refers to *objective* risk assessment as compared to RP which is subjective. Objective risks can be statistically estimated (e.g., the calculation of probability and estimated damages of environmental disasters). Similar to RP, the time frame, scope, and certainty of a threat can vary in risk assessment. Here, risk is conceptualized as the product of probability and consequences of an undesired event (Yuan et al. 2009).

*Risk communication* is the field of research that deals with the exchange of information and education about risk-related content. Risk communication is relevant to a wide range of contexts (e.g., avoiding industrial accidents, illnesses, traffic, disasters) (Wogalter et al. 1999; Fischhoff 1995). Its importance lies in the fact that successful risk communication can lead to improved safety behavior without having to learn from experience. For the case of fire evacuation, risk communication may contribute to an increased awareness and preparedness of occupants as well as to more effective evacuation behavior. Risk communication affects RP.

*Safety climate* refers to a (work or living) community’s shared perception of their organization’s policies, procedures, and practices as they relate to the value and importance of safety within the organization (Griffin and Neal 2000). Safety climate may affect RP and other factors such as situation awareness.

*Safety culture* summarizes the shared values and beliefs in an organization which interact with its structures and control systems to produce safety related behavioral norms (Thompson et al. 1996). Similar to safety climate, safety culture influences RP.

*Arousal* refers to the general activation of the sympathetic nervous system. Although not directly related to RP, arousal may be strongly correlated with the underlying physiological and psychological processes of RP. For example, arousal affects decision-making, in the sense that higher arousal is related to more impulsive decision-making (Strack and Deutsch 2004).

*Fear* is an emotional response to a perceived threat and a common reaction to emergency situations (Öhman 2000). Animal studies sometimes use fear reactions as an indicator of RP (Stankowich and Blumstein 2005).

### Theoretical frameworks on RP and evacuation

Since RP is relevant to multiple disciplines, several theoretical frameworks addressing RP have been developed. Nonetheless, very few studies on RP during evacuation mention being founded on a specific theory or theoretical framework (See Table 1). The following sections introduce theoretical models related to RP and human behavior in emergency situations. Most of the theories follow the *psychometric paradigm*, which is the basis of the risk-as-feeling approach (Slovic 2000) and aims to develop objective, reliable and valid measurement tools of psychological processes (e.g., rating scales or standardized questionnaires) (Eignor 2013). Within the psychometric approach, quantitative subjective ratings of perceived and acceptable risk are mapped together with the desired level of control for a given situation and compared to ratings of other situations (Slovic 1987).

#### Heuristic-systematic models

The *Heuristic-Systematic* approach refers to two-process models of information processing and can be applied to RP. Such models are widespread in the psychology literature (e.g., Strack and Deutsch 2004; Chaiken and Maheswaran 1994; Chaiken and Eagly 1989; Chaiken 1980; Kahneman 2011). The basic assumption is that information can be processed systematically, heuristically, or in a combination of the two. In systematic information processing, all available information is assessed according to its meaning and relevance. This process is thorough but also demands more time and resources. *Prospect theory* (Kahneman and Tversky 1979) first introduced the concept of heuristics, which can be understood as mental shortcuts or simple rules of thumb that allow for the making of fast decisions at the cost of less systematic information processing. Heuristics are useful tools for decision-making if sufficient information about probabilities or time and other resources for a slower and more thorough assessment are not available. For example, research on natural disasters has shown that the actual probability of an event is rarely regarded in risk appraisals (Miceli et al. 2008). Several types of heuristics are relevant for evacuation and RP:

The *affect heuristic* refers to the fact that current emotional states influence decision-making. This concept is an important part of the risk-as-feeling approach. Emotional states modulate the understanding of numbers and probabilities. For example, large numbers are underweighted in decisions and lack meaning for people unless they convey a feeling (Slovic 2010b). Similarly, judgments of risk and utility are often influenced by whether one likes something, e.g., utility is overestimated and risk underestimated for activities associated with positive emotions (Slovic et al. 2005, 2007; Finucane et al. 2000).

*Anchor Heuristics* describe the tendency to overly rely on a few initial or salient pieces of information (anchors) when making a decision. Subsequent or less salient cues may be ignored or weighed less. Anchoring itself can be affected by mood, expertise, or the use of other heuristics (Furnham and Boo 2011). This may lead to over or underestimation of risk during fire evacuation. For example, an occupant might interpret the sound of a fire alarm as a cue for a drill and subsequently ignore more subtle cues of a real incident.

*Availability heuristics* describe how likelihood estimates of an event are affected by how easy it is to recall or imagine it (Kahneman and Tversky 1979). The more “available” an event is in memory, the higher its estimated likelihood (Greening et al. 1996). For example, occupants may assess the risk of a fire emergency by the ease of recalling similar occurrences.

*Representativeness heuristic* notes that likelihood estimates of an event are often judged by their similarity to its parent population (Kahneman and Tversky 1979). The more a cue seems to fit into a certain category of events, the more likely it will be estimated as indicative of it. For example, occupants may perceive a fire alarm sound as not indicative for a fire emergency if it sounds similar to other alarm sounds (e.g., an error sound from an electronic device).

Similarly, *proximity heuristics* describe the “tendency to judge probabilities by monitoring the spatial, temporal, or conceptual distance to a target” (Teigen 2005, p. 424) and has been studied to understand estimates of accident probabilities. An example of a proximity heuristic might be the case of occupants overestimating the probability or severity of a fire emergency if they perceive fire cues matching their expectations about a fire emergency scenario.

The use of heuristics may explain another type of bias known as *normalcy bias*, which refers to the tendency to interpret cues as indicative for everyday events and underestimating the likelihood and consequences of disasters (Okabe and Mikami 1982). During building fires, when occupants are faced with ambiguous information, the normalcy bias is likely to last longer while occupants remain inside the building (Kuligowski and Gwynne 2010). Additionally, the anchor, availability and representativeness heuristics may lead to low perceived risks, since many fire cues are not specific to an emergency. For most cases, the assumption that a fire alarm is just another drill and not a real emergency is true. During the evacuation of the World Trade Center (WTC) on September 11, 2001, occupants, especially those from the lower floors, reported relatively low RP which may be attributed to the assumption that nothing extraordinary was going on in the building (McConnell et al. 2010).

When processing information *systematically*, individuals aim to understand the available information and its relevance for RP and decision-making. This process is relatively slow and requires significant resources. In heuristic information processing, RP is based on relatively automatic processes in which little effort is spent on processing the information (Smerecnik et al. 2012).

Whether information is processed systematically or heuristically depends on an individual’s level of arousal (i.e., activation of the sympathetic nervous system), available cognitive resources, and other factors, such as experience, emotional states, or personality traits (Strack and Deutsch 2004). Perceived risk, as defined above, may also determine whether information is processed heuristically or systematically. In line with this, a study by Sherman et al. (2011) on building evacuation suggested a curvilinear relationship between perceived risk and information seeking behavior, an indicator of systematic processing. Participants reporting either low or high perceived risk were less likely to seek more information as opposed to those with a medium level of perceived risk (Sherman et al. 2011). Both systematic and heuristic processes can lead to an evacuation decision, but they may be affected by different factors. Both processes are prone to biases and limited within each individual.

Similar to the systematic and heuristic approach, the concept of *bounded rationality* describes that decision-making is limited by the available information, the cognitive resources, and the finite amount of time to make a decision. Here, the term *satisficing*, describes the process in which occupants do not base their decisions on all available information but on the amount of information they deem sufficient for their decision (Simon 1972, 1991). Satisficing could be understood as a form of heuristic information processing. Drabek developed the *stress–strain perspective* based on the concept of bounded rationality (Drabek 2001). In line with the heuristic-systematic approach, constraints (e.g., the availability of information) in the social and physical environment are hypothesized to bias RP and behavior during emergencies.

#### Appraisal models

Several psychological models underline the importance of appraisal processes for decision-making. The most prominent of these models is the *Transactional Stress Model*, which is not a RP model per se but provides insights on coping mechanisms when people are faced with risk (Lazarus and Folkman 1984). It is a classic cognitive theory on emotion regulation, is closely linked to RP, and postulates several appraisal processes.

- Primary appraisal: “How relevant is this situation to my needs?” Is there the risk of harm, loss, threat, or challenge? In the case of evacuation, this is the assumed reaction to the alarm signal. If the alarm is deemed relevant, the next appraisal process follows.
- Secondary appraisal: “Do I have the necessary resources available to cope with the situation?” If yes, then problem-focused attempts to cope with the situation are used. If no, then emotion-focused coping is used (i.e., If I cannot change the situation, I have to adapt my emotional reaction to it).
- Re-appraisal after coping attempts: “How is the situation now?”

Problem-focused coping does not automatically imply that occupants would choose adequate reactions. Classic cognitive stress models, such as the transactional stress model, focus on the subjectively perceived threat of a situation (Lazarus and Folkman 1984), which can be interpreted as RP. Psychological stress occurs if one does not possess the necessary resources to cope with a situation which is perceived as dangerous.

Appraisal processes, similar to the ones discussed in the *Transactional Stress Model*, have been incorporated into theories developed specifically for human behavior in fire. Proulx’s cognitive stress model of people facing fire, for example, takes into account different factors, such as information processing, decision-making, problem-solving, and stress (Proulx 1993). Similar to the Transactional Stress Model, Proulx sees iterative appraisals of the situation and one’s own coping resources at the core of experienced stress and behavior. According to this model, several *stress loops* are triggered when occupants are confronted with a fire outbreak, in which the appraisal of ambiguous information and increased danger can lead to fear, worry, and confusion (Proulx 1993).

The importance of appraisal processes during catastrophic events has been empirically studied. For example, in a questionnaire study with hurricane survivors, Riad, Norris, and Ruback found that 58% of the respondents chose not to evacuate from a severe hurricane threat. The most important reasons for not evacuating during a hurricane were that the hurricane had not been perceived as a serious threat (primary appraisal), participants had been confident that their current place was as safe as any other (secondary appraisal), and participants avoided thinking about the situation (coping, Riad et al. 1999). The misinterpretation of valid threat indicators may therefore be a key problem in the process of evacuation. Evidence from a hypothetical scenario study showed that participants appraised different types of disasters (crime, natural disaster, terrorist attack) as similar in risk, but they differed in the intentions to take protective actions. For example, in a natural disaster scenario participants were more likely to state they would change their daily activities than in a crime scenario (Heilbrun et al. 2010). The cognitive appraisal of a given situation as dangerous may influence the readiness to engage and the choice of protective actions. For example, a recent meta-analysis showed that the motivation to participate in safety trainings rises if the consequences of a potential event are perceived as threatening (Burke et al. 2011a).

#### Protective action decision model

The *Protective Action Decision Model* (PADM) was developed to provide a holistic approach to human behavior in emergency situations. It sets up a descriptive framework of the information flow and decision-making that affects protective actions taken in response to disasters (Lindell and Whitney 2000; Lindell and Perry 1992; Kang et al. 2007; Huang et al. 2012; Houts et al. 1984; Kuligowski 2011). The model describes the path from the initial perception of hazard cues to the initiation of protective action. It takes a variety of predispositions, such as environmental or social context, into account. Furthermore, it stresses the importance of appraisal processes, and thus links cognitive psychological approaches, such as the aforementioned transactional stress model, with classic safety engineering models.

A brief overview of the processes in the PADM follows with a discussion of the role of RP in the model. For a more comprehensive description of the model, see Kuligowski (2011); (Kuligowski 2013). PADM differentiates between pre-decisional and decisional processes. The former are the basis on which an individual makes his/her evacuation decision. The pre-decisional processes comprise (1) perceiving, (2) directing attention to, and (3) comprehending relevant fire cues. If the three pre-decisional processes have identified potentially relevant fire cues, occupants are hypothesized to engage in a five step decision-making process which may result in protective actions (Lindell and Perry 2012; Perry and Lindell 2004):

1. Risk identification: Is there a real threat that I need to pay attention to? [If yes, then the occupant believes the threat]
2. Risk assessment: Do I need to take protective action? [If yes, then the occupant decides that he/she needs to take protective action]
3. Protective action search: What can be done to achieve protection? [The occupant begins searching for possible protective action strategies]
4. Protective action assessment: What is the best method of protection? [The occupant chooses one of the action strategies developed in the previous stage and develops a protective action strategy or plan]
5. Protective action implementation: Does protective action need to be taken now? [If yes, the occupant follows the plan developed in the previous stage]

As stated earlier, RP is defined in this review as the subjective evaluation of the probability to be affected by an imminent undesirable event and the assessment of one’s own perceived vulnerability. This corresponds to the two first decisional processes in PADM. Lindell and Perry incorporate threat perception into PADM, which can be understood as an equivalent to primary appraisal in the transactional stress model (Lindell and Perry 2012; Lazarus and Folkman 1984). Their approach to RP corresponds to the expectancy-value approaches discussed earlier and also includes emotional and motivational aspects (labeled dread and unknown risks) (Lindell and Perry 2012). The first stage of the decision model involves hazard identification in which the properties of a potential threat have to be evaluated. In the second step, risk assessment, ones’ own vulnerability toward the threat is judged. This clearly represents the cognitive side of RP (systematic assessment of expectancy and values). One may speculate that the *risk-as-feeling* side is at least implicitly part of the pre-decisional as well as the risk identification and assessment processes.

It is also possible to integrate heuristic processing into PADM. Heuristics could play two different roles in the PADM. First, heuristics and systematic processing may be competing at each decisional step. Depending on the arousal, cognitive resources, previous experience, or the result of one of the decisional processes, an occupant may process each of the five decisional questions heuristically or systematically. For example, an occupant who identified a potential risk and sees him or herself as extremely vulnerable may rely on heuristics to identify protective actions. Second, heuristics may lead to skipping some of the decisional processes and thus speed up the decision-making at the cost of less thorough reasoning. Consider, for example, the anchor heuristic. An occupant might interpret the sound of a fire alarm as a cue for a fire emergency and immediately start evacuating without going through the steps of protective action search and assessment.

It is important to understand how RP affects evacuation activities. As theorized by the PADM, RP can be understood as a threshold mechanism for evacuation decision-making (Siebeneck and Cova 2012; Kates 1971). Therefore, it is possible to hypothesize that there is a threshold of acceptable risk for an occupant before he/she decides to evacuate. Evacuation decision-making is “triggered” if the perceived risk becomes unacceptable.

The PADM is a descriptive model of decision-making during emergency situations. As such, it does not make predictions about future behavior. However, it is possible to integrate the processes described in the PADM into predictive models, such as the *Evacuation decision model (EDM)*. EDM aims to predict the point in time when the decision to take protective action is made and assumes that RP is the key factor in this process (Reneke 2013).

#### Reasoned actions models

**Figure 2:**
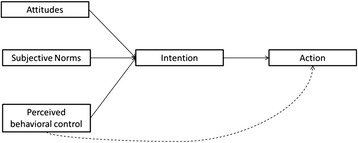
**The Theory of planned behavior (TPB); Redrawn from Rogers & Prentice-Dunn** (1997)**.**

*Reasoned actions models*, such as the Theory of Planned Behavior (TPB, Figure 2) or the Theory of Reasoned Action (TRA), are general theories describing how intentions are transferred into actions (Ajzen 2011; Sheppard et al. 1988). They fall into the systematic branch of the heuristic-systematic approach. These models assume that “intentions are the immediate antecedents of behavior and intentions themselves are a function of attitude toward the behavior, subjective norm, and perceived behavioral control” (Ajzen 2011). RP plays a role in an individual’s assessment of his/her perceived behavioral control (i.e., Do I have the resources to change the odds for an undesired event?). Most applications of these models have been used to predict long term behavior (e.g., changes in health behavior). However, it seems possible to apply the TPB to planned evacuation behavior. One study applied the TRA to hurricane evacuation behavior (Kang et al. 2007). TRA assumes that occupants’ conscious intentions to engage in a behavior are the principal determinants of actual behavior. The main limitation of this approach is that it is purely cognitive and leaves out affective situational variables (e.g. fear and anxiety). However, unanticipated barriers can arise between the intention and the opportunity to act, thus making the actual behavior different from the behavioral intention (Fishbein 1979).

A meta-analysis of protection motivation models showed that increases in threat severity, threat vulnerability, response efficacy, and self-efficacy facilitated adaptive intentions or behaviors. Decreases in maladaptive response rewards and adaptive response costs also increased adaptive intentions or behaviors (Floyd et al. 2000).

The *Protection Motivation Theory* (Rogers and Prentice-Dunn 1997) is a model developed to understand and predict long-term health behavior. It tries to explain the effects of threatening (health) information on changes in attitude and behavior (e.g., planning to quit smoking after learning about the smoking related diseases). Protection motivation theory can also be applied to (planned) evacuation behavior. It hypothesizes that perception of the severity, susceptibility, or probability of occurrence, perceived self-efficacy, and perceived response efficacy modulate protective actions (Cauberghe et al. 2009).

Although the reasoned action models were not developed to understand building fire evacuation, they have important similarities with other models (e.g., PADM) and may help to better understand RP and evacuation behavior. Unlike the more disaster specific models, the reasoned action models have been studied and found applicable to a wide range of behaviors. Thus we speculate that at least for planned evacuation, similar processes like the ones described in TRA or TPB can be assumed. However, these models do not apply to spontaneous and unplanned behavior and mostly ignore situational and affective variables. The important question is whether building fire evacuation is planned behavior or not. The answer to this question has consequences on how RP has to be conceptualized. If evacuation was a predominantly planned behavior, RP would most likely have to be understood from an expectancy-value perspective. If evacuation behavior does not involve long term planning, the risk-as-feeling approach may be more relevant. For most occupants, evacuation is clearly not a long term planned behavior in the sense that occupants plan the following: “When the fire alarm goes off, I will (not) evacuate”. However, the time from the initial cue to the evacuation decision can be seen as a planning phase (as it is in the PADM) in which occupants appraise their situation, vulnerability, resources and options. Future studies should investigate to what degree evacuation from an imminent threat is planned behavior.

#### Hazard to action chain model

**Figure 3:**
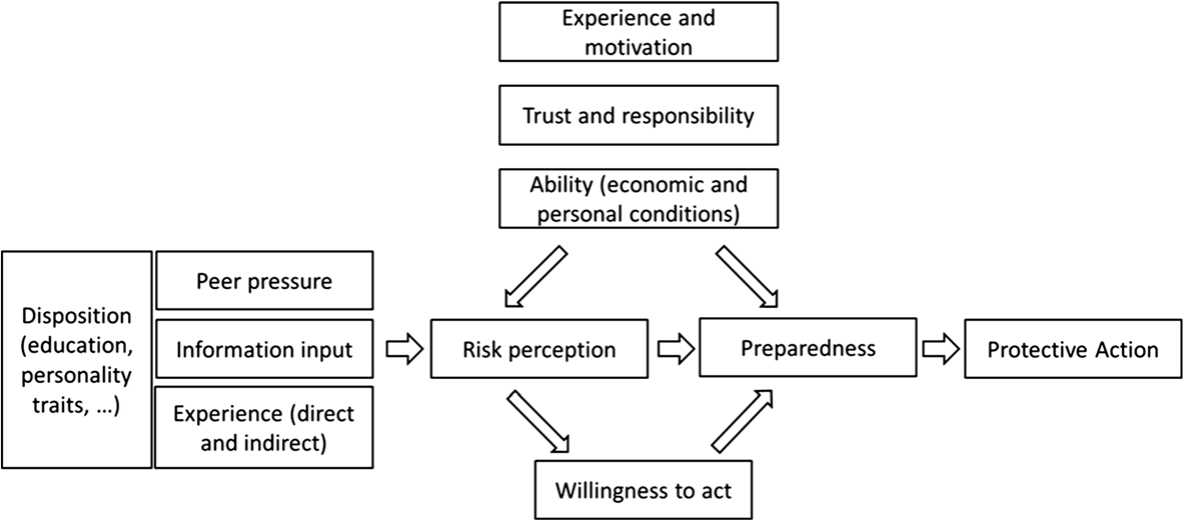
**The hazard to action chain; Redrawn from Wachinger et al.** (2012)**.**

Wachinger et al. (2012) developed the *Hazard to action chain model* (Figure 3) based on a literature review describing the effect of RP on protective actions during natural disasters. The authors assume that, similar to reasoned action models, intentions (labeled as “willingness to act”) and preparedness are the precursors of (protective) actions. According to this model, RP influences preparedness as well as intentions: The higher the perceived risk, the higher the preparedness and intentions. The authors found that the most robust predictors of RP were trust in authorities (lower trust leading to higher perceived risk) and previous personal experience of a natural disaster.

Unlike the models discussed before, this model makes few assumptions about the actual decision-making process. However, the authors hypothesize that high trust in authorities could be seen as a form of heuristic processing. Individuals may trust authorities when they are confronted with complex and unknown hazards which require swift decision-making. Further research is required to show whether this model is also applicable to fire emergencies.

#### Security motivation system

RP is also studied from an evolutionary perspective. Understanding the biological side of RP can help to develop theories on human behavior and decision-making. Life threatening events, such as fires, are experienced only rarely. Furthermore, indicators of a potential threat are often not easily detectible or may be ambiguous. The question is how organisms adapt to threats that may not occur in every generation. Woody and Szechtman propose a *security motivation system* (SMS) as part of the central nervous system, designed to adapt the organism to extremely rare life threatening events (Szechtman and Woody 2004). The SMS detects “subtle indicators of potential threat, to probe the environment for further information about these possible dangers, and to motivate engagement in precautionary behaviors, which also serves to terminate security motivation” (Woody and Szechtman 2011). The authors postulate that the SMS is represented in hardwired neural circuits and its activation motivates protective actions through increased arousal and vigilance, enhanced detection of threatening cues, and the facilitation of future behavioral responses to such cues (Hinds et al. 2010). Applied to the situation of fires, cues such as the smell of smoke or other people moving to an emergency exit may activate the SMS.

The SMS can be integrated into other theoretical concepts. The SMS has two major functions: (1) to detect and process threat relevant cues and (2) to trigger protective action when a threat is detected (Trower et al. 1990; Woody and Szechtman 2013). These functions correspond closely to the assumption about the pre-decisional processes in PADM or the risk-as-feeling approach. The highly automated functions of the SMS can be seen as precursors of the decisional processes in PADM. Woody and Szechtman applied the SMS to policy making (mainly dealing with information about terrorist threats) (Woody and Szechtman 2013). The authors argue that the SMS is triggered by information about life-threatening events and not by abstract threats, regardless of the actual probability of the event. This offers a potential explanation for cognitive biases or the use of heuristics in emergency situations.

#### The mediator hypothesis

All theoretical frameworks reviewed so far, assume that RP is in some way causally related to evacuation decisions (See also Section What role does perceived risk play in building fire evacuation?). However, evacuation decision-making could be possible without feeling at risk. According to the aforementioned *mediator hypothesis,* RP mediates between individual factors and actual risk reduction behavior. The idea of this hypothesis is that an evacuation decision is not solely dependent on the outcome of an RP process. In fact, it assumes that evacuation is also possible without perceiving risk. Some occupants may evacuate simply because they received instructions to do so. For example, occupants who experienced several fire drills previously may follow instructions to evacuate assuming that these are part of yet another drill and not perceive fire related risk.

Some studies support the assumptions of direct and indirect pathways between individual factors and evacuation decisions. In a study on wildfire risk behavior (note that this is not immediate evacuation behavior), Martin et al. (2009) found that risk reducing behavior during wildfires was associated with individual factors such as subjective knowledge and locus of responsibility mediated by perceived risk. In the same study, self-efficacy, defined by Bandura (1997) as the extent or strength of one's belief in one's own ability to complete tasks and reach goals, had a direct (non-mediated) effect on risk reduction behavior. In addition, perceived risk was clearly associated with risk reduction behavior (Martin et al. 2009). Another study found that perceived risk mediated the effect of gender on evacuation from flood but not wind events (Bateman and Edwards 2002). Future studies should aim to disentangle the causal relationships between perceived risk and evacuation decision-making.

## What role does perceived risk play in building fire evacuation?

In this section literature is presented on how RP affects evacuation behavior and on factors influencing RP itself. Models, such as the PADM discussed in the previous section, state that occupants need to appraise whether a situation provides a threat before they decide to take protective action. However, one may also speculate that RP is not necessarily a precursor of evacuation and that there may be cases in which occupants begin egress without necessarily feeling at risk as the mediator hypothesis suggests. With that said, there are several hypothetically possible links between RP and a protective action decision:

1. RP directly causes protective action decision-making and behavior. High perceived risk leads people to engage in protective actions (e.g., evacuation or defend in place), whereas low perceived risk may even lead to non-protective actions (e.g., delaying, actively ignoring cues).
2. RP may affect evacuation decision-making and behavior but other factors do so as well.
3. RP is a mediator and it accounts for the relationship between a predictor variable (e.g., other human factors) and protective action decision-making (See Section The mediator hypothesis).
4. RP is a moderator and it affects the direction and/or strength of the relationship between a predictor variable and protective action.
5. RP is a correlate of protective action decision-making and behavior but not a causal factor.
6. RP may be independent of evacuation decision-making (i.e., occupants may feel not at risk and evacuate or feel at risk and not evacuate).

Although some of these potential links are mutually exclusive, it is possible that different links are operating in parallel or at different stages of the evacuation process (e.g., in the pre-evacuation and the evacuation period). Future research is necessary to identify which of the potential links are the most important interrelations of RP and protective actions.

### RP during the World Trade Center evacuation on September 11, 2001

A significant amount of research on RP and evacuation has been published on the attacks on the World Trade Center (WTC) on September 11, 2001. Several independent studies found that perceived risk was positively correlated with the likelihood to make evacuation decisions and faster response times, and low perceived risk was associated with delayed evacuation (Day et al. 2013; Gershon et al. 2007; Kuligowski 2011; Sherman et al. 2011). Kuligowski (2011) developed a predictive model of evacuation decision-making based on qualitative interviews with evacuees from the WTC on September 11, 2001. The author found that perceived risk fluctuated throughout the evacuation process (Kuligowski 2011). Based on the PADM, RP, in the form of risk identification and assessment, was found to play an important role in predicting protective action identification, assessment and implementation (i.e., the decision to evacuate). Some building occupants felt at risk immediately after they noticed initial cues of the impact in WTC 1 (*rapid perceivers*), causing them to decide earlier than others to evacuate. However, in some cases, the perceived risk of the rapid perceivers decreased after a while, as they received additional cues that the author categorized as less intense. Other occupants who received less intense cues, or more intense cues later on in the event, felt at risk only at a later point (*late perceivers*) or not at all (*non-perceivers*). RP was only found to trigger evacuation, if the risk was personalized. Gershon et al. (2012) reported that 70% of the interviewed WTC occupants stated that feeling at risk triggered their *evacuation decision*. That is, these results support hypothesis 2 stating that RP may be one important but not the only factor influencing the decision to evacuate.

A closer look reveals an even more complex situation as some of the mentioned studies on the 2001 WTC disaster also found seemingly contradictory results. Sherman et al. (2011) studied *evacuation delays* during the attack on the WTC on September 11, 2001. The authors operationalized perceived risk in a single item rating as the perceived “seriousness” of the situation. Here, higher perceived risk was found to be connected to shorter evacuation delays and fewer pre-evacuation behaviors (which again shortened evacuation delays, in line with hypothesis 1, 2, or 5). Sherman et al. (2011) also found that higher perceived risk may also lead to more information seeking behavior which in turn prolonged evacuation delays (Hypothesis 3 or 4). In another study on the evacuation from WTC on September 11, 2001 by Kuligowski and Mileti (2009), RP was operationalized as a yes/no question on whether occupants believed that somebody else had been killed in the event. Here, higher perceived risk was correlated with more information seeking, more pre-evacuation actions, and longer evacuation delays. However, the path-analysis performed in that study revealed that RP was not directly connected to evacuation delays after controlling for the number of cues, the floor level, information seeking, and the number of pre-evacuation actions (Hypothesis 3). In that model, RP predicted information seeking behavior and the number of pre-evacuation actions in WTC2 (Kuligowski and Mileti 2009). Sherman et al. (2011) suggest a curvilinear relation of RP and information seeking behavior, i.e., if the perceived risk is either extremely high or low, occupants are less likely to seek more information (see also heuristic-systematic approach). Note that differences in the operationalization of RP, data collection and the samples may explain the differences between the studies. The data presented in Sherman et al. (2011) was collected several months later than the data from Kuligowski and Mileti (2009), and the sub-sample reporting the highest perceived risk in Sherman et al. (2011) was not included in Kuligowski and Mileti (2009). Kuligowski and Mileti report that perceived risk was rated at four points in a narrative interview process: when the first cues at WTC 1 impact where noticed, at the point the decision to evacuate was made, when participants noticed the WTC 2 impact, and when participants realized that WTC 2 had collapsed. Sherman et al. (2011) used a questionnaire format (paper pencil and web based) and the rating of perceived risk was administered independent of a narrative flow. With regard to the definition of RP given earlier, it is not clear to what extent the items used in both studies measured perceived risk. Whereas the item in Sherman et al. (2011) could be understood as an evaluation of one’s own vulnerability, Kuligowski and Mileti’s rating could have been understood as an implicit measure of perceived probability. These differences underline the importance of a clear definition of RP and standardized, reliable, and valid (construct validity, Cronbach and Meehl 1955) measures of RP.

Another indicator of the complexity of the problem was demonstrated in a study by Day et al. (2013) on RP during evacuation from WTC on September 11, 2001. In this study, participants were asked to rate their perceived risk on a seven point Likert scale during different stages of the evacuation process. The authors found a significant negative correlation between perceived risk and response time (Hypothesis 5). However, they also reported that several participants did not give ratings of perceived risks, as they reported not remembering to have evaluated their risk (Hypothesis 6) (Day et al. 2013). Note that simply not remembering having assessed the risk of a situation does not imply that these occupants did not feel at risk. It is possible, for example, that memory effects biased the participants’ responses. Nonetheless, these findings indicate that at least two of the possible links between RP and protective actions mentioned earlier – correlation (5) or independent (6) – are possible.

In summary, the studies discussed here draw a complex picture of the role RP during evacuation from the WTC on September 11, 2001. Some of the differences in the results of the studies may be attributed to the fact that the studies by Sherman et al. (2011), Kuligowski and Mileti (2009), as well as Day et al. (2013) used different data sets and operationalized RP differently. Given the retrospective correlational nature of the studies, however, an investigation of a potential causal relationship between perceived risk and evacuation behavior is not possible.

### Further evidence and open questions

Although the role of RP during evacuation is still inconclusive, one may speculate how RP affects evacuation decision-making. McGee and Russell found that the personalization of risk is an important link between awareness of a hazard and mitigation actions (McGee and Russell 2003). This finding is in line with the theoretical framework models discussed above (e.g., PADM). According to the heuristic-systematic modeling approach, the level of perceived personal risk affects the level of information processing. Systematic processing can be most likely expected at moderate levels of perceived risk; whereas heuristic processing is expected at either low or high levels. As already mentioned, this may then determine how occupants move through the decisional processes suggested in the PADM.

The question still remains as to how significant RP is to evacuation behavior. Or more specifically, how much variance in evacuation behavior can be explained by RP? For example, Riad et al. (1999) found a number of highly significant correlative associations between RP and evacuation (in line with Hypothesis 5), but the reported effect sizes were relatively small. All in all, perceived risk improved the prediction whether someone would evacuate by 8% compared to chance (Riad et al. 1999). Unfortunately, not many studies on RP and evacuation report effect sizes (See Table 1).

Wachinger et al. (2012) propose three hypotheses to explain why some studies on natural disasters did not find a connection between RP and protective action. All three hypotheses introduce moderator variables which also seem potentially applicable to building evacuation. Although the meaning assigned to RP during evacuation from latent threats, such as disasters, may be different, it is possible to develop similar hypotheses for building fire evacuation:

1. Occupants perceive high risks but do not decide to engage in protective action because they think that staying in place outweighs the estimated subjective costs of protective action (e.g., having to stop one’s work, not wanting to make a fool of one self, or expecting difficulties while evacuating). This could be of particular importance if there are competing motives (e.g., one’s own safety vs. property attachment).
2. Occupants perceive high risks but trust that others, foe example authorities, will help them. This hypothesis may be less relevant for building evacuation from an acute threat. However, it underlines the importance of credible evacuation communication by authorities.
3. Occupants perceive high risks but do not think they have sufficient resources to engage into protective actions (e.g., mobility impaired occupants may not be able to use stairs for evacuation). Again this underlines the importance of credible evacuation communication and/or instructions. Occupants need to know their options in order to engage in protective action.

Future studies are clearly necessary to understand the role of RP in building fire evacuation. These studies should investigate (1) the causal relationship between RP and evacuation decision-making and behavior, as well as (2) underlying reasons why some occupants do not evacuate although they may feel at risk.

### Factors potentially modulating RP

Several factors potentially modulate RP which can be broadly differentiated into situational, individual, social, and organizational factors (Table 2). Some of these factors are dynamic, in that they may change during the course of an emergency situation, (e.g., available fire cues or emotional states) and some are static (e.g., context or previous experience). Furthermore, these factors interact with each other and can be affected by the RP process itself. For some of the static factors, distributions can be either assumed or derived from the literature. For example, the factor *prior experience* could be operationalized as the percentage of occupants in a building who have previously experienced an event or evacuation.

**Table 2:** **Current knowledge on factors affecting perceived risk and evacuation behavior**

**Factor**	**Category**	**Static/dynamic** ^**1**^	**Effect on perceived risk**
Fire Cues	Situational	Dynamic	More, closer, unexpected and more intense fire cues lead to higher perceived risk
Hazard proximity	Situational	Dynamic	Inconclusive
Floor level	Situational	Dynamic	The higher the floor, the higher the perceived risk
Context	Situational	Static	Inconclusive
Credibility of information	Situational	Static	Credibility of information moderates information processing and perceived risk with potential interaction effects of the source of information (another person vs. system)
Complexity of information	Situational	Dynamic	Inconclusive
Gender	Individual	Static	Tendency toward lower perceived risk in men, but effects are potentially modulated by age and context
Age	Individual	Static	Inconclusive
Previous experience	Individual	Static	Direct effects of previous experience on perceived risk are inconclusive.
Behavioral training	Individual	Static	Inconclusive
Hazard knowledge	Individual	Static	Knowledge about hazards increases perceived risk
Property attachment	Individual	Static	Inconclusive
Personality traits	Individual	Static	Inconclusive
Emotional states	Individual	Dynamic	High arousal and state anxiety increase perceived risk
Medical factors	Individual	Dynamic	Inconclusive
Cognitive abilities	Individual	Static	Inconclusive
Information Processing	Individual	Dynamic	Information that is processed easily may be associated with lower perceived risk
Trust in authorities	Individual	Static	High trust reduces perceived risk; low trust increases perceived risk
Cognitive bias	Individual	Dynamic	Inconclusive
Behavior of others	Social	Dynamic	Behavior of others moderates the link between perceived risk and protective action
Social roles	Social	Dynamic	Inconclusive
Groups	Social	Dynamic	Higher perceived risk in groups
Organizational context	Organizational	Dynamic	Inconclusive

*Note:* ^**1**^Dynamic factors can change in the course of a fire emergency, e.g., the number of fire cues may increase or decrease with time.

References for the findings are given in the text in Section 4.4.1 to 4.4.4.

As the role of RP during fire evacuation is complex (see above), the factors potentially influencing RP also interact with each other and affect other important variables in the evacuation process. For example, the number and intensity of cues and the floor level affected not only RP, but also had a direct impact on pre-evacuation delays in two studies on the WTC evacuation on September 11, 2001 (Kuligowski and Mileti 2009; Sherman et al. 2011). The exact interaction among RP, evacuation decision-making and evacuation delay is still not entirely clear (e.g., what is a mediating or a moderating variable? Which factors are mainly correlates but have effects on evacuation behavior independent of RP?). Findings on several of the factors identified as being connected to RP and evacuation are described below.

#### Situational factors

Situational factors refer to all aspects of circumstances at a given moment that influence RP and/or evacuation. These cues originate mainly from the physical environment of an occupant.

*Fire cues* refer to all cues initiated by a fire^1^. Fire cues that are greater (in number), closer in proximity, and more intense have been linked to higher perceived risk (Sherman et al. 2011; Kuligowski and Mileti 2009; Kuligowski 2011). In addition, sudden and unexpected cues may increase perceived risk in the sense that unusual, surprising events produce cues which occupants cannot identify immediately (Sherman et al. 2011).The accuracy of information conveyed by fire cues is relevant, especially since information that clearly and unambiguously indicates threat can increase perceived risk. This may explain why studies found that poorly designed alarm systems may not induce high enough perceived risk in occupants (Caroly et al. 2013; Kuligowski 2011).

*Hazard proximity* refers to the spatial proximity of occupants to a threat; its role on RP is still inconclusive although research suggests that it may modulate perceived risk. Some studies found that the higher the perceived risk, the closer the hazard was to occupants (Kuligowski and Mileti 2009), while other studies did not find this effect (Fahy and Proulx 2002). It is possible that other factors, such as the relative location of occupants to the hazard and known exit routes modulate the effect of hazard proximity. Visibility, vertical and horizontal distance, or other factors, may be important confounding factors. In addition, it is not clear if the relation between hazard proximity and perceived risk is, for example, linear or non-linear.

*Floor level* refers to the absolute floor level of an occupant in a building, irrespective of his/her distance to the fire (which would be measured by the *hazard proximity* factor mentioned above). The current state of research concludes that perceived risk increases with floor level in high-rise buildings. In the case of a full building evacuation, the *floor level* in a high-rise building was a significant predictor of perceived risk during the evacuation from the WTC on September 11, 2001 (the higher the floor, the more perceived risk) in one study but not in another (Sherman et al. 2011; Kuligowski and Mileti 2009). Although future studies should investigate whether the absolute floor level or the position relative to the fire origin (*hazard proximity*) is more relevant.

*Context*, broadly defined as the general circumstances of an event, affects human behavior in fire in several ways and its effect on RP is still not fully understood. Preparedness, vigilance, and the interpretation of fire cues may vary over different contexts (e.g., public events, workplace, or home setting). A questionnaire study demonstrated that participants’ self-reported perceived risk varied over different settings (e.g., financial vs. safety related decision-making) (Weber et al. 2002). The underlying mechanism may be that environmental cues are interpreted differently over different contexts. In a residential home, for example, occupants may perceive cues about a fire from the fire itself or from smoke detectors. In public buildings most occupants may only receive information from the fire alarm system (e.g. through public announcements). Another explanation may be that cognitive biases (e.g., normalcy bias), social roles and perceived responsibility, availability of emergency procedures (e.g. evacuation plans), and the interpretation of cues may vary across contexts.

*Credibility of information*^b^ refers to the perceived level of credibility that a person assigns to a piece of information or source of information. Overall, this factor moderates information processing and RP with potential interaction effects of the source of information. Credibility of risk-related messages affects information processing (see, heuristic systematic approach) as well as RP and has been extensively studied in the context of disaster preparedness (Mileti and Sorensen 1990). One study on long term RP showed that risk assessment after receiving risk-related information was in part mediated by heuristic and systematic information processing. Here, highly credible sources were associated with more heuristic information processing and lower perceived risk. In turn, low credibility of an information source is associated with more systematic information processing and higher perceived risk. If the source of information was an industry or government organization, higher credibility was correlated with lower perceived risk scores in this study. If the source of information was another person, however, this correlation was inverted (Trumbo and McComas 2003). Another study on hurricane evacuation showed that people use different sources of information and their trust in the credibility of these sources varies (Lindell and Whitney 2000).

The *complexity* of a situation, including the information provided to building occupants in a fire situation, may affect whether information is processed systematically or heuristically. In one basic research study, participants rated ostensible food additives as more harmful when their names were more difficult to pronounce than when their names were easier to pronounce. The study indicates that information which is more demanding to process increases perceived risk (Song and Schwarz 2009). Transferring that to evacuation scenarios, Drabek hypothesizes that inconsistency, ambiguity and overload of information increase emergent perceived risk (Drabek 2001). In line with these findings, another study found that mobility impaired building occupants associated lack of information (ambiguity) with highest ratings of concern during a fire evacuation (McConnell and Boyce 2013). However, given the small number of studies on the effect of the complexity of a situation on RP, further research is clearly necessary.

#### Individual factors

Individual factors refer to factors within a person that may affect RP and evacuation behavior. These can be either state (i.e., dynamic, for example, emotional states or arousal) or trait (i.e., stable, for example, gender, age, cognitive abilities) variables.

##### Gender

Lower perceived risk (Slovic 2010a) and less risk-averse attitudes (Weber et al. 2002) of men compared to women might explain gender differences in evacuation behavior. However, no influence of gender on risk identification and assessment was found in Kuligowski’s analysis of evacuation decision-making during the WTC disaster on September 11, 2001 (p. 148) (Kuligowski 2011). In Sherman et al.’s WTC evacuation study, being female was associated with increased perceived risk (Sherman et al. 2011). A meta-analysis found that men were more likely to engage in risk taking behavior, but that this effect was modulated by context (i.e., the kind of threat) and age (i.e., with growing age, the differences seemed to get smaller) (Byrnes et al. 1999). In another study, gender differences in RP could be explained by differences in self-reported fear and anger (Lerner et al. 2003). In summary, men seem to perceive less risk than women.

*Age* is correlated with several evacuation-relevant variables (e.g., experience, cognitive and physical abilities, education, social role, etc.); however, its role with regard to RP is still inconclusive. Some authors argue that older adults are better in risk evaluation than younger adults since they have to practice risk-related decisions more frequently in their daily life (e.g., medication labeling, adaption to changes in physical fitness) (Wilson et al. 2013; McLaughlin and Mayhorn 2014). This is in line with research on driving behavior, which states that deficits due to reduced physical abilities or reaction times can be compensated on a strategic and tactical level (higher vigilance). Further research is needed since some studies found that older occupants are less likely to evacuate (Riad et al. 1999) but others found no relation between age, perceived risk, and evacuation delay (Sherman et al. 2011; Kuligowski and Mileti 2009; Kuligowski 2011).

*Previous experience* with fire emergencies or similar situations may significantly affect RP, vigilance, and preparedness and has been identified as one of the strongest predictors of increased perceived risk during natural disasters (Wachinger et al. 2012). However, experiencing a disaster without experiencing personal harm may decrease perceived risk. (For an overview of studies on RP, experience, and natural disasters, see Wachinger et al. 2012). Research from volcano disasters showed that having experience in a disaster diminished differences in RP between volcano experts and untrained participants (Bird and Gisladottir 2012). Similarly, survivors of the 1993 WTC bombing had shorter evacuation delays than occupants who had no such experience during the evacuation of the WTC on September 11, 2001 (Day et al. 2013). Therefore, the effects of previous experience on RP are still under debate, although it seems possible that increased perceived risk moderates the connection between evacuation decision-making and previous experience.

*Behavioral training* aims to convey behavioral or theoretical knowledge through practice. Although the effects of behavioral training on RP is still unclear, it is known to improve evacuation behavior (e.g., Kinateder et al. 2013). The ability of novice drivers to detect hazards can be improved through training, as one study showed that trained participants scanned the environment for potential threats more frequently and efficiently than the control group (Pradhan et al. 2006). That is, training may increase *preparedness* and *vigilance* for fire cues, and the effectiveness of training depends on the severity of perceived risks (Burke et al. 2011a).

*Hazard knowledge* refers to the knowledge that any person has related to specific types of hazards associated with an incident, including the consequences of the hazard and appropriate responses. This factor has been shown to increase perceived risk, although these effects are complex and still not fully understood. In line with studies showing that knowledge is correlated with the adoption of risk reduction behaviors (Lindell and Whitney 2000; Shields et al. 2009), Kuligowski and Mileti found that obtaining additional information after receiving initial fire cues was weakly correlated, but with statistical significance, with perceived risk during the evacuation from WTC on September 11, 2001 (Kuligowski and Mileti 2009). However, unknown or ambiguous events are also associated with increased perceived risk (Song and Schwarz 2009). In turn, *familiarity* with an event reduces perceived risk (Riley 2014), although familiarity does not necessarily imply knowledge. An overuse of warnings and false alarms may consequently lead to a desensitization of occupants and may reduce their perceived risk during a real emergency (Rando et al. 2007). Further research is necessary to disentangle the effects of hazard knowledge and familiarity on RP.

*Property attachment* or *territorial functioning* may not directly affect RP, but may mitigate the connection between perceived risk and evacuation (see also *Context*). In studies on hurricane evacuation, homeowners reported that a reason for not evacuating was the fear of looting (i.e., perception of risk to personal property; Riad et al. 1999; Kang et al. 2007; Huang et al. 2012). In some cases, occupants returned to their desk to pick up personal items during evacuation of WTC on September 11, 2001 (Kuligowski 2011). Further research is necessary to clarify the effects of property attachment on RP.

*Personality traits* refer to relatively stable “patterns of thoughts, feelings, and actions in a diverse array of psychological phenomena, including motives, wishes, apperceptions ^c^, and attitudes, as well as behaviors in which a person processes information (Mccrae and Costa 1995).” Although future studies need to clarify the exact role of personality traits and RP during fire emergencies, some personality traits, such as impulsivity or sensation seeking, are related to risk taking behavior and may be important for RP (Zuckerman and Kuhlman 2000; Ryb et al. 2006). Based on personality traits, individuals may vary in how they perceive risk. Highly impulsive occupants, for example, may require a lower number of fire cues to perceive a high enough risk before they decide to take protective actions. One study found that the relation between personality traits and risky driving behavior was mediated by risk-related attitudes (Ulleberg and Rundmo 2003).

*Emotional states*, such as state anxiety, are correlated with arousal (the activation of the sympathetic nervous system), and can increase perceived risk. Higher arousal is associated with more impulsive information-processing (Strack and Deutsch 2004) and may bias RP. High state anxiety affects the way that hazard cues are processed and reduces cognitive resources (Mathews and MacLeod 1985; Yiend 2010). Emotional states may also affect the readiness with which cues are interpreted as threatening and an attentional bias on threatening stimuli (Cisler and Koster 2010). One questionnaire study on terrorism-related hazards found that risk appraisals were modulated by fear and anger. Highly fearful participants reported higher perceived risk, and participants scoring high on the anger scale also reported lower perceived risk (Lerner et al. 2003).

*Medical factors* (including intoxication) affect how fire cues are perceived and information is processed, but the effects on RP are still inconclusive. For example, alcohol intake distorts RP in the sense that that it modulates arousal and may lead to more risky behavior (Mongrain and Standing 1989). However, the range of medical factors potentially modulating perceived risk is vast and future studies are necessary.

*Cognitive abilities* refer to the ability to understand fire related cues. Although still inconclusive, some cognitive impairments, such as mild cognitive impairment or dementia (Christensen et al. 2013; Brown et al. 2012) may reduce the ability to perceive and understand fire related cues and reduce the ability to perceive risk as well as to comply with evacuation procedures. So far, there are no studies that directly address RP and cognitive abilities in the context of evacuation and future studies are necessary to understand their role during evacuation.

*Information processing* and RP potentially interact; however, the exact relation is still inconclusive. Basic research shows that low processing fluency (i.e., the ease with which information can be processed) fosters the impression that a stimulus is unfamiliar, which in turn results in perceptions of higher risk (Song and Schwarz 2009). That is, higher cognitive load when processing unfamiliar information is associated with higher perceived risk. Empirical studies on information processing and RP during fire evacuation are necessary to verify whether these results can be transferred to emergency situations.

High *trust in authorities* reduces perceived risk (and may even lead occupants to underestimate a hazard in unprotected areas), whereas low trust (or distrust) increases perceived risk (Wachinger et al. 2012). The authors further note that trust in authorities can be understood as a heuristic supporting decision-making in complex situations and when facing unknown or ambiguous threats. Trust in authorities, similar to credibility of information (see above), may also mediate the path between perceived risk and protective actions. Furthermore the authors hypothesize that damaging trust may increase perceived risk (Wachinger et al. 2012). Another hypothetical scenario study found a correlation between the degree of trust in authorities and hazard appraisals for certain technological risks (Siegrist and Cvetkovich 2000). As most of the research on trust in authorities and RP focusses on evacuation from natural disasters, further research with regard to fire evacuation is necessary.

*Cognitive biases* refer to systematic distortions in human information processing and decision-making. The use of heuristics may lead to such biases.

- *General RP bias*: In general, perceived risk of events is correlated with the actual risk. However, there are some biases in the sense that small risks are overestimated and high risks are underestimated (Sjöberg 2000; Lichtenstein et al. 1978; Thompson and Mingay 1991; Mbaye and Kouabenan 2013).
- *Positivity bias (comparative optimism)* refers to the fact that occupants consistently rate their own personal risk as lower than the risk to others. This phenomenon is well documented in the literature, and was found in one study on tunnel fire emergencies (Sjöberg 2000; Horney et al. 2010; Mbaye and Kouabenan 2013).
- *Locus of control/perceived control*: risks perceived to be under one’s own control are more acceptable than risks perceived to be controlled by others. Illusion of control was found to be correlated with perceived invulnerability (positivity bias) and negatively with perceived risk in a study on accidents in chemical and nuclear facilities (Mbaye and Kouabenan 2013).
- *Normalcy bias* reduces perceived risk and refers to a tendency to attribute cues to ‘normal’ events during disasters and not to catastrophic events. During the evacuation of the WTC on September 11, 2001 occupants, especially from the lower floors, reported relatively low perceived risk which may be attributed to the assumption that nothing extraordinary was going on in the building (McConnell et al. 2010).

#### Social factors

Social factors mainly refer to the effect of others on one’s own RP and behavior. This can be broadly labeled as social influence. Social influence is defined as changes in attitudes, beliefs, opinions or behavior as a result of the fact that one is confronted with the attitudes, beliefs, opinions, or behavior of others (Hewstone and Martin 2008).

##### Behavior of others

The behavior of others potentially moderates the link between RP and protective action. Seeing other occupants evacuate provides a cue for an emergency and may increase personal perceived risk. In turn, passive behavior of others may trigger the normalcy bias (i.e., that nothing is wrong) and reduce perceived risk. Alternatively, social influence may lead occupants to ignore their own appraisal of the situation. Studies from the evacuation of a cinema theater showed that the non-evacuation behavior of others could thwart evacuation (Nilsson and Johansson 2009; Kinateder et al. 2014a, b). Social influence on RP may be a function of knowledge, as one study showed that experts, in comparison to untrained participants, rely less on information derived from others (Siegrist and Cvetkovich 2000). Further studies testing the specific relationship of social influence, perceived risk and protective action are necessary.

The effect of s*ocial roles* on RP is still inconclusive. However, it is possible to assume that social roles affect RP as a function of perceived responsibility and knowledge. For example, trained fire wardens may improve their hazard detection skills and be more vigilant. In turn, the behaviors of fire wardens, or occupants with assigned authority, may influence the perceived risk of other occupants. Survivors of the WTC attacks reported that being told to evacuate by others (especially people in fire safety roles or roles of authority) triggered their evacuation decision (See also *Social trust*) (Gershon et al. 2012; Kuligowski 2011). Further studies are also necessary to investigate the impact social roles have on the scope of an occupants’ perceived risk (See 3.1 Scope of RP). For example, one may speculate that occupants whose social role includes high perceived responsibility for others (e.g., a fire fighter or a parent) extend the scope of their RP to others.

Occupants in *groups* may experience higher perceived risk than occupants who are alone during a fire emergency. During the evacuation from WTC 1 on September 11, 2001, occupants who grouped together during the event reported higher perceived risk (Trumbo and McComas 2003). However, only one study was found to provide evidence of this linkage. Therefore, future studies are necessary to test if occupants with higher perceived risk are more likely to form groups, or whether forming groups increases perceived risk.

#### Organizational factors

Organizational factors refer to the effects of the organizational structure on RP during evacuation. In a study on the September 11, 2001 WTC evacuation, participants working for the New York/New Jersey Port Authority reported higher perceived risk during the incident (Sherman et al. 2011). One may speculate that an organization’s safety climate and culture affects RP and social roles, which in turn influences protective action of the organization’s member. A qualitative study of the WTC disaster on September 11, 2001 showed that evacuation was affected by worksite preparedness planning, including the training and education of building occupants as well as risk communication (Gershon et al. 2007).

##### Organizational context

Depending on where an emergency occurs (e.g., work, home, public places; see also *context*), RP and evacuation behavior may be different. In one questionnaire study participants reported higher compliance rates to hypothetical evacuation orders if they were at work than at home (Dombroski et al. 2006). One reason might be that occupants felt safer or perceived less risk in their home environment. Depending on the organizational context, the perceived risk necessary before an evacuation decision is made might be different.

#### Summary of factors

Table 2 summarizes the findings regarding the individual factors potentially affecting RP during emergencies. A literature review on RP during natural hazards (e.g., evacuation from hurricanes or floods) concluded that the previous experience and lack of trust in authorities had the strongest direct effects on RP (Wachinger et al. 2012). Future studies are necessary to test whether this holds true for building fire evacuations as well. Similarly, further research is clearly necessary regarding all the factors identified in the present review, since so many of the relationships were inconclusive (See Table 2).

## Overview of studies

Table 1 gives an overview of studies used in this review. The studies are sorted according to their relevance to RP during building fire evacuation. A comparison of the studies reveals that there are very few studies on RP in the context of a building fire evacuation.

## Limitations

There are some limitations to the present review. The literature reviewed for this report varies significantly in nature and scope. The question of scope, or what to include and what not to include in this review, was therefore an important one (Ogilvie et al. 2005a, b). The theoretical models selected and discussed here focus on RP as the process of an individual occupant. There are additional theories that address risk and RP in other contexts, e.g. in strategic decision-making (e.g., Zheng and Cheng 2011; Lo et al. 2006) or as a social or organizational phenomenon such as the social amplification of risk framework (Kasperson et al. 1988). However, these theories are beyond the scope of the present article.

Another issue is publication bias. Generally speaking, very few studies on RP report results where no correlations were found between RP and evacuation. This indicates that there might be a publication bias towards positive relations between RP and evacuation. The Cochrane collaboration and other researchers have repeatedly shown that studies with significant and positive results are easier to find than those with non-significant or 'negative' or null results (Guyatt et al. 2011). This may have caused an over-representation of studies finding correlations between RP and evacuation.

As already mentioned previously, the bulk of the literature on evacuation and RP relies on self-reported rating scales. Many authors operationalized RP with 1 item questions such as “How ‘at risk’ did you feel at particular moments during the evacuation process?” (e.g., Kuligowski 2011; Day et al. 2013; Martin et al. 2009; Siebeneck and Cova 2012; Matyas et al. 2011; McConnell et al. 2010; Horney et al. 2010). Single items are an easy to use and economical approach to measure RP. However, the question(s) may not grasp RP in its full complexity and it is possible that participants had different concepts about what they meant when they rated their perceived risk. Using different methods of measurements may lead to significantly different outcomes, as demonstrated by the comparison of two studies on evacuation from the WTC on September 11, 2001 (Sherman et al. 2011; Kuligowski and Mileti 2009). Furthermore, it is crucial that the measurement tool actually reflect the construct of interest (construct validity). It is questionable if a single item can validly measure a construct that consists of two independent dimensions (perceived probability and vulnerability). However, single item measures can have sufficient predictive validity (Wanous et al. 1997; Robins et al. 2001; Elo et al. 2003). It is necessary to develop and test measuring tools (e.g., questionnaires) for RP during building fire evacuations that meet common quality criteria for objectivity, reliability, and validity (Eignor 2013) of psychometric testing.

Most of the data about RP and evacuation reported here relies on self-report data. As previously noted, the understanding of the term risk perception may vary greatly within the population. Self-report studies are extremely useful to get an understanding of occupants’ experiences and behaviors during evacuation. However, self-report data are prone to bias due to social desirability and other sources of bias (e.g., memory effects). That is, self-reported behavior or behavioral intentions may differ greatly from actual behavior. Slovic (2010a) reports the case of a study in which participants were asked whether the construction of a nuclear power plant would stop them from using an adjacent beach. Most participants stated that they would stop using the beach if the plant was built. The power plant was built, and no decline in the attendance of the beach was observed.

In the present review, the authors discussed the effect of several factors on RP. In each case, an attempt was made to identify correlations or causal effects between each factor and RP, individually. However, RP in complex situations, like a fire emergency, is most likely determined by multiple variables, which may interact with each other. That is, the conclusions of the present review may oversimplify how various factors increase or decrease perceived risk and may neglect potential interaction effects between the factors.

The present review on the role of RP during fire evacuation is heavily based on studies of one single event (i.e., the attacks on WTC on September 11, 2001). Although these studies revealed comparable results using independent databases, and knowledge on RP has significantly advanced based upon these research efforts, it stands to reason that the events of September 11, 2001 may not allow for generalization to all other building fires. Future studies are necessary to build a broader database. Such studies should take into account different contexts (e.g., with regard to occupancy or location). Only a limited number of studies were found using data from laboratory settings or drills. Additionally, prospective studies are extremely scarce in this field (Table 1). The development of ecologically valid and ethical laboratory paradigms for the study of evacuation and RP may prove especially useful.

Finally, this literature review depended on the accessibility of sources. This review is limited to the libraries of the National Institute of Standards and Technology and the University of Würzburg, Germany. For literature without full text access from either of these two libraries or through interlibrary loan, abstracts were considered, or the source was ignored.

## Conclusions and outlook

The first goal of this overview was to clarify the concept of RP in the context of building fire evacuation and to provide a definition of RP specifically for this field. RP was defined and differentiated from other similar concepts, such as situation awareness. In this paper, RP is seen as a psychological process comprising the subjective evaluation of the probability to be affected by an imminent threat and an assessment of one’s own perceived vulnerability and coping resources. It is modulated by affects and prone to cognitive biases. In a second step, the following relevant theoretical frameworks on RP from evacuation research were identified and described: Heuristic-systematic approach, PADM, Transactional stress model, reasoned action models, and SMS. We believe that this synopsis may contribute to theory development in the field of evacuation research.

In a next step, factors potentially influencing RP during building fire evacuation were identified and discussed. The results of this discussion, summarized in Table 2, revealed that the number of fire cues, floor level in high-rise buildings, credibility of information, gender, previous experience, hazard knowledge, certain emotional states, information processing, certain cognitive biases, the behavior of others, and groups can affect RP.

Future research will have to clarify the relationship of the factors identified in the present review. Specifically, three future research steps are necessary: (1) Development of a self-report questionnaire of RP for fire evacuation that meets common quality criteria of psychometric testing. The variety in which perceived risk was measured in the studies reviewed in the present article indicates that a common standard to study RP during fire evacuation is necessary. An objective, reliable, and valid questionnaire is necessary to understand RP during fire evacuation. (2) Identification of specific effects of perceived risk during the pre-alarm and the protective action phase of a fire emergency. Controlled laboratory studies which systematically manipulate RP could shed light on how perceived risk influences RSET. One possibility could be to manipulate the expectation and emotion component of perceived risk through conditioning (e.g., by presenting aversive stimuli combined with a scenario (context information)) or priming (e.g., by presenting information about fire emergencies) and then confront participants with a hypothetical emergency scenario. Another possibility would be to compare the reaction to fire cues of participants who differ systematically in trait RP (e.g., by comparing reactions of highly fearful and non-fearful participants). (3) Development of a holistic predictive model on the interaction of the factors potentially modulating RP. Although the present review identified a set of factors that most likely influence perceived risk during fire evacuation, it is unclear how strong the effects of individual factors are and how these factors interact with each other.

The present review demonstrates that RP is relevant to evacuation outcome variables such as evacuation decision-making and evacuation delays. We introduce a definition of RP during fire evacuation, allowing a more precise operationalization of the concept. A precise operationalization of RP potentially allows researchers to explain additional variance in occupants’ evacuation decision-making and behavior, and, consequently, may improve the prediction of ASET/RSET in engineering tools.

## Endnotes

^a^In the present article, the term *fire cue* refers to all cues provided in a scenario initiated by a fire. These are not restricted to fire effluent cues and include indirect indicators of a fire emergency (e.g., seeing other occupants evacuating or receiving information via a public address system).

^b^Here, the authors are describing the effect of information credibility on risk perception. Whereas this is inconclusive, other research, as well as NIST guidance, suggests that credibility of information has an important influence on evacuation decision-making, response, and protective action behavior (Kuligowski and Omori 2014).

^c^Apperception in the psychology literature refers to consistent patterns on how people perceive their environment in relation to their past experience.
